# Parental doubts regarding childhood vaccinations after the COVID-19 pandemic: a qualitative study from Poland

**DOI:** 10.3389/fpubh.2025.1606815

**Published:** 2025-07-30

**Authors:** Sandra Janiak, Elwira Piszczek, Krzysztof Buczkowski

**Affiliations:** ^1^Department of Family Medicine, Collegium Medicum in Bydgoszcz, Nicolaus Copernicus University in Torun, Bydgoszcz, Poland; ^2^Institute of Sociology, Nicolaus Copernicus University in Torun, Torun, Poland

**Keywords:** vaccination hesitancy, adverse effect following immunization, parental attitudes, qualitative study, vaccine safety, healthcare communication

## Abstract

**Background:**

Vaccinations are one of the most effective methods of preventing infectious diseases. Data published in recent years indicate a decline in routine childhood vaccination rates. Vaccine hesitancy is an increasingly serious problem, recognized as one of the 10 most significant threats to global health.

**Object:**

This study aims to identify the causes of parental hesitancy regarding childhood immunizations after the COVID-19 pandemic.

**Methods:**

We conducted 33 in-depth interviews with parents who were hesitant to vaccinate their children. We analyzed the data using qualitative content analysis.

**Results:**

The main themes identified included concerns about adverse events following immunization, unsatisfactory communication with healthcare providers, distrust in the recommended vaccination schedule, individual risk assessment, conspiracy theories/anti-system sentiments, and organizational/financial barriers.

**Conclusion:**

The leading causes of vaccine hesitancy were fear of adverse events following immunization and unsatisfactory communication with healthcare professionals. These findings underscore the urgent need for improved training in communication, empathy, and negotiation skills among healthcare providers, along with the delivery of consistent, evidence-based vaccine information across all levels of care.

## 1 Introduction

Vaccinations are one of the most effective ways to prevent infectious diseases. Currently, they prevent 2–3 million deaths annually, and an additional 1.5 million could be avoided if global vaccination coverage improved (1). Despite this, many regions worldwide have seen a decline in vaccination rates. In July 2022, WHO and UNICEF published data showing the most significant decline in routine childhood immunization in 30 years. A decreased perception of the importance of childhood immunizations was reported in 46 out of 55 surveyed countries (in data covering the years 2015–2022) (2). As a result, diseases that could be eradicated through widespread immunization continue to appear. A clear example is the doubling of measles cases among children in 2022 compared to the previous year (2). The global coverage for the first dose of the measles vaccine fell to 81% in 2021, the lowest level since 2008. This means that in 2021, 24.7 million children missed their first dose of the measles vaccine — 5.3 million more than in 2019. Furthermore, 14.7 million children missed the necessary second dose (3).

The emergence of polio in Israel, the United Kingdom, and the United States in 2022 further highlighted that even significant progress in combating diseases could be at risk if vaccination rates decline (2).

Vaccine hesitancy (VH) is a primary driver of this decrease. This term refers to the delay in acceptance or refusal of vaccines despite the availability of vaccination services. Recognizing its growing impact, the World Health Organization (WHO) identified vaccine hesitancy in 2019 as one of the 10 most significant threats to global health (1). In 2015, the Strategic Advisory Group of Experts on Immunization (SAGE) proposed a definition of VH as a complex and context-specific phenomenon influenced by factors such as complacency, convenience, and confidence (4). They ultimately accepted the term “hesitancy” to describe a continuum of attitudes toward vaccination, ranging from strong anti-vaccine positions (complete refusal without any doubts) to full acceptance, with individuals who are more or less hesitant falling in between (4–6). The problem of declining vaccination rates and increasing hesitancy was further exacerbated by controversies surrounding newly introduced vaccines, particularly COVID-19. Moreover, attitudes initially directed solely at COVID-19 vaccines quickly spread to other vaccinations. Discussions about vaccine safety took place in the media, online, in doctors’ offices, and even during family gatherings (7).

Researchers have extensively examined the growing erosion of vaccine confidence and the broader expansion of vaccine hesitancy in the context of the COVID-19 pandemic. A study in the United Kingdom highlighted that the decline in vaccine confidence following the pandemic was particularly evident among younger adults (8). While these individuals represent the next generation of parents, concerns about vaccination are already highly relevant among current caregivers. Childhood vaccination plays a crucial role in preventing the spread of infectious diseases and safeguarding both individual and public health, making it a significant focus. Understanding the attitudes of today’s parents-who make immunization decisions for themselves and their children-has become a vital area of research. The urgency of this research grows as more parents actively seek information and critically assess medical recommendations rather than passively follow routine childhood immunization schedules (9). Unfortunately, the sources they rely on are not always reliable, contributing to the growth of anti-vaccine attitudes (9). Such attitudes are further reinforced by distrust in politicians, pharmaceutical companies, and even healthcare workers, prompting parents to seek information about vaccines online (10). This information is often inaccurate and contradicts established medical knowledge. The issue of VH is complex and multidimensional and must be studied within historical, cultural, and economic contexts (11).

Poland has historically been one of the countries with very high childhood vaccination rates (12, 13). This was primarily the result of parents passively complying with mandatory vaccination programs at a time when individual rights were severely restricted. These attitudes gradually shifted with the democratization of the political system and the Westernization of lifestyles, and parents began to play a more active role in vaccination decisions (14). This has led to a noticeable downward trend in vaccination rates. According to some analyses, lower social capital and trust in public institutions typical of postcommunist countries may contribute to reduced trust in vaccinations and vaccination policies (15, 16). As in other countries, understanding the arguments put forward by parents who refuse vaccinations — and even more importantly, those who are hesitant about vaccinating their children — is key to building trust in childhood immunization (17). A review of the available literature revealed a lack of qualitative research conducted after the outbreak of the COVID-19 pandemic that explores parents’ concerns and attitudes toward childhood vaccinations (18). In Poland, existing studies on vaccine hesitancy have primarily relied on standardized research methods (such as surveys and questionnaires) and quantitative statistical analyses (19).

Therefore, we decided to conduct a qualitative study to obtain a more comprehensive understanding of this issue. This study aims to identify the causes of parental hesitancy regarding childhood immunizations after the COVID-19 pandemic in a population with historically high childhood vaccination coverage.

## 2 Materials and methods

### 2.1 Design

A qualitative inquiry method was employed to establish an empirical foundation for understanding the perspectives of parents who were hesitant to vaccinate their children (20). Following the definition of vaccine hesitancy proposed by SAGE, we interviewed parents who exhibited behaviors such as delaying vaccinations, selectively accepting vaccines, or refusing them, while expressing uncertainty about their decisions (5). We conducted in-depth, semi-structured interviews, which facilitated detailed exploration of participants’ views and created a setting that encouraged openness. The individual format of the interviews allowed participants to feel comfortable, share personal experiences, and express their opinions freely (21).

### 2.2 Participants, setting, and data collection

This study is part of the qualitative segment of a broader project investigating parents’ doubts and attitudes toward childhood vaccinations.

The study was conducted between December 2023 and July 2024, reaching data saturation by the end of this period. Participants were recruited using purposive sampling from parenting forums on Facebook, organizations in contact with the target group (such as clinics, nurseries, kindergartens, and schools), and the “snowball” method, where participants shared information about the study through word-of-mouth, phone calls, or text messages.

The study included parents who were hesitant and who had at least one child under the age of 18. Both biological parents and legal guardians with permanent caregiving responsibilities were considered parents in this research.

The topic guide was developed, tested, and refined through a pilot study with four individuals who met the inclusion criteria. Interviews were conducted in Polish and followed a semi-structured topic guide. Each interview began with an introductory question followed by prompts, as outlined in Table 1.

**Table 1:** Topic guide for interviews.

Opening question	What do you think about vaccinations?
Prompt 1	What do you think about vaccinating your children?
Prompt 2	Do you have any negative experiences with vaccinations?
Prompt 3	What do you think about new vaccines? Are they less safe or safer than the old ones?
Prompt 4	Do you believe that natural immunity gained after recovering from a disease is more effective than the immunity gained through vaccination?
Prompt 5	Do you believe that all vaccinations should be voluntary? Or is it good that some vaccinations are mandatory?
Prompt 6	What do you think about adverse vaccine reactions?
Prompt 7	Where do you get your information about vaccinations? Which sources do you think are the most trustworthy?
Prompt 8	Do you discuss your children’s vaccinations with others? If so, with whom (family, close friends/strangers) and where (online/offline)?
Prompt 9	What most significantly influences your decision to vaccinate or not vaccinate?

A total of 11 interviews were conducted in person, and 22 were conducted via phone. All interviews were conducted by a single researcher trained in qualitative research methods. The participants had no prior relationship with the researcher. Before each interview, the researcher introduced herself and explained the purpose of the study. Written consent was obtained for participation and recording. Participation was voluntary, and participants could withdraw at any time.

Interviews lasted between 15 and 45 min, were audio-recorded, transcribed verbatim, and then anonymized. No interviews were repeated, and participants did not review the transcripts or comment on the results.

Data saturation was reached after the 25th interview; however, additional scheduled interviews were still conducted to ensure no new themes emerged and to enhance the robustness of the findings.

### 2.3 Data analysis

This study employed qualitative content analysis as outlined by Graneheim and Lundman (22), Sandelowski (23), and Hsieh and Shannon (24) to organize and describe the data. This approach provided a transparent, descriptive summary of the semi-structured interview data.

The analysis involved several steps: identifying meaning units, creating condensed descriptions, coding, and identifying subthemes and themes. A team of three researchers conducted the study.

Two authors (SJ and EP) independently analyzed the transcripts, identifying meaning units. These units were condensed and labeled with general descriptive codes (Table 2). The codes were then compared and grouped into subthemes and themes.

**Table 2:** Examples of meaning unit, condensed meaning unit, interpretation, subthemes and themes, from content analysis.

Meaning unit	Condensed meaning unit, description close to the text	Interpretation (Code)	Subtheme	Theme
“I doubt my child is allergic to chicken egg protein. These vaccines are produced on some chicken embryos, right? (...) And I hope nothing happens, that he will not have, so to speak, an allergic reaction.”	“I doubt my child is allergic to chicken egg protein. These vaccines are made using chicken embryos. I hope he will not have an allergic reaction.”	Parental concern about potential allergic reaction to vaccines due to egg protein.	Parental about immediate effects – allergic reactions after vaccination.	Concerns about AEFI.

Subsequently, SJ, EP, and a third researcher (KB) held two meetings to discuss and refine the definitions of the subthemes and themes until a consensus was reached. Findings were reported using the Consolidated Criteria for Reporting Qualitative Research (COREQ) (25).

### 2.4 Ethical considerations

Ethical approval for this study was obtained from the Bioethical Committee of the Collegium Medicum in Bydgoszcz at Nicolaus Copernicus University in Torun, Poland (KB 476/2023). Potential participants were informed about the details of the study, and those who chose to take part provided informed consent. Both confidentiality and anonymity were guaranteed to all participants.

## 3 Results

Among the recruited parents, 23 participants were mothers, and 10 were fathers (Table 3). Most respondents (54.5%) lived in large cities, held higher education degrees (75.8%), and were employed at the time of the study (72.7%). The parents’ ages ranged from 23 to 59 years, with a median age of 38.06. Participants had between one and four children, with the majority (57.6%) having two children. Seven participants reported that their child had a chronic illness.

**Table 3:** Sociodemographic characteristics of parents (*N* = 33) and children.

	*N*	%
Gender		
Female	23	69,7
Male	10	30,3
Place of residence		
Big city	18	54,5
Small city	7	21,2
Village	8	24,2
Employment status		
Employment	24	72,7
Unemployment	9	27,3
Education level		
Tertiary^a^	25	75,8
Secondary^b^	5	15,2
Basic vocational/sectoral	1	3,0
Completed primary	1	3,0
Unknown	1	3,0
Age^c^		
18–25	1	3,0
26–35	11	33,3
36–45	17	51,5
46–55	3	9,1
55–60	1	3,0
Number of children		
1	8	24,2
2	19	57,6
3	5	15,2
4	1	3,0
Children’s age^d^		
At birth up to 1	8	12,7
1–2	12	19,0
3–4	9	14,3
5–6	8	12,7
7–10	14	22,2
11–18	7	11,1
18 + years	5	7,9
Chronic illness of a child^e^		
Does not occur	26	78,8
Occur	7	21,2
Mandatory childhood vaccinations ^e^		
All	22	66,7
Only some	7	21,2
None	4	12,1

^a^Including: master’s degree, bachelor’s (licentiate) and persons holding a college graduation diploma. ^b^Including: vocational secondary education and persons with post-secondary education. ^c^Mean age was 38.06 years (range 23–59 years, SD = 6,9). ^d^Data calculated for 63 children. ^e^Calculated for 33 respondents surveyed.

The analysis identified six main themes containing several subthemes (Table 4). In many cases, these themes overlapped. Below is a description of each theme and subtheme, with participant quotes indicated by participant numbers.

**Table 4:** Themes and subthemes.

1. Adverse Event Following Immunization	Concern about the long-term effects of vaccinations, mainly neurological complications, weakening of natural immunity Concern about immediate effects – allergic reactions after vaccination Feeling left alone when adverse events occur Lack of compensation Difficulty in reporting (also: not knowing how to do it) Personal/family/friends’ experiences/hearsay Expectation tests and consultations before vaccination to reduce the risk of adverse events
2. Communication with medical staff	Brief, routine qualification examinations, not tailored to the individual (including too short consultation times) The need for an empathetic approach from doctors, as parents with doubts do not want to be stigmatized Disagreement among experts – one doctor recommends, another advises against a particular vaccine Perception that doctors do not have the proper knowledge – lack of trust in medical personnel’s competence
3. Distrust of the recommended vaccination schedule	Too many vaccines in general (also: too many in a short time) Vaccines should be administered at a later age Combining multiple vaccines in one visit Lack of flexibility in the medical staff’s approach to individualizing vaccination schedules (rescheduling/postponing/delaying vaccinations)
4. Risk assessment	Mild illness vs. adverse events Rare diseases vs. adverse events Chronic illness in a child – complications from VPD vs. adverse events after vaccination
5. Conspiratorial thinking/Anti-system sentiments	Lack of trust in the government and pharmaceutical companies Differences in the content of foreign (original) and Polish-language leaflets for the same vaccine Concern about the composition of vaccines Lack of voluntary choice (or rather, mandatory vaccination) Lack of comparative studies between vaccinated and unvaccinated children Too rapid introduction of vaccines to the market
6. Organizational and financial issues	Higher-quality vaccines that public funds do not cover Lack of information about optional additional vaccines (e.g., chickenpox) Lack of reminder information from primary care providers about vaccination requirements

### 3.1 Adverse event following immunization

#### 3.1.1 Concern about the long-term effects of vaccinations

Fear of side effects was the most commonly cited barrier to vaccination. Neurological complications were the primary concern for parents. These included developmental delays, seizures, and potentially epilepsy. An intense fear was also expressed regarding autism, with many parents convinced that there was a link between vaccines and the development of autism.

*“Yes, Doctor, of all the vaccine side effects, I fear neurological complications the most.” (Participant 16).*

*“An unvaccinated child will not have seizures... seizures only occur in vaccinated children.” (Participant 19).*

*“With the MMR vaccine and others, there is some risk of autism in children.” (Participant 17).*

However, while concern about neurological AEFI (adverse event following immunization) was prevalent, some parents were more skeptical about the vaccine-autism link. Some explicitly stated that they did not believe vaccines caused autism:

*“No, I know a lot about autism. Vaccines do not cause autism.” (Participant 21).*

Another primary concern for parents was the belief that vaccines weaken children’s natural immunity. It is known that respiratory infections are more common in children than in adults, partly due to exposure in daycare and kindergarten. However, some parents interpreted their children’s frequent infections as a negative consequence of vaccination:

*“Before vaccination, the kids were healthy. After vaccination, they caught every little infection. After the measles, mumps, and rubella vaccine, my daughter got strep throat four times.” (Participant 10).*

One participant noted that her child became healthier after stopping flu vaccinations:

*“I feel like ever since I stopped vaccinating him for flu, he has not gotten sick at all.” (Participant 16).*

Parents also feared that some adverse effects might appear only after a long period. Developmental disorders and illnesses emerging in early childhood, such as autism, febrile seizures, or sudden infant death syndrome, were often temporally linked to childhood vaccinations. Parents were convinced there were causal relationships, raising doubts about vaccine safety:

*“I know that many AEFIs show up later — not right after the shot, but months or years later.” (Participant 15).*

#### 3.1.2 Concern about the immediate effects of vaccinations

Although most participants focused on what they considered serious side effects, some also highlighted potential allergic reactions to vaccines. These concerns were mainly expressed by parents whose children had known allergies:

“*I have concerns because my child is allergic to egg protein. These vaccines are produced using chick embryos, right? So I hope nothing happens — no allergic reaction.” (Participant 23)*

#### 3.1.3 Feeling unsupported in navigating post-vaccination concerns

Another significant concern was handling AEFI cases and the support offered to parents whose children experienced side effects. Parents felt abandoned and unsupported. They acknowledged that medicine cannot predict everything, but they expected doctors to take responsibility if an adverse event occurred:

*“...the child could even die. The doctor confirms that, but when we ask if they will take responsibility and put that in writing, they refuse.” (Participant 4).*

*“Parents are simply scared because they are the ones left with a disabled child, not the doctor.” (Participant 10).*

One participant shared the story of a fellow anti-vaccine activist whose three out of five children experienced severe vaccine side effects, which reinforced the perception that AEFIs are common. Moreover, these parents received no support:

*“I have a friend from the ‘STOP NOP’ (STOP AEFI) group. I did not know she was in it until I asked why. It turned out that three of her five children had severe vaccine side effects, and she did not get any help. That’s where it all started.” (Participant 25).*

#### 3.1.4 Gaps in the financial support system

When discussing support after AEFIs, participants primarily referred to financial compensation:

*“As far as I know, there have been court rulings in the United States awarding significant compensation to families whose children developed illnesses within two or three years after vaccination. Unfortunately, we do not have that system here.” (Participant 15).*

#### 3.1.5 Physician-parent dynamics in adverse event reporting

A legal issue raised during the study was the reporting of AEFIs. In Poland, AEFIs can be reported by healthcare professionals and patients or their guardians. Parents expected their child’s doctor to file the report, and failure to do so was perceived as ignorance of the connection between symptoms and vaccination:

*“Doctors do not always want to report it, even though they are required to. They usually say there’s no connection.” (Participant 22).*

*“We never got our AEFI officially recognized because our pediatrician insisted there was no connection to the vaccine — that it just happened.” (Participant 26).*

#### 3.1.6 Subjective reports from individuals and their close contacts

Sharing personal experiences greatly influences the credibility of vaccine-related narratives and is a crucial determinant of vaccine attitudes. Stories of children’s adverse reactions, often detailed with photos, videos, or medical documentation, were considered highly credible by participants. Parents noted that even rare events, when described vividly, significantly increased their fear of similar outcomes for their children:

*“Immediately after vaccination, he had a very high fever, his leg hurt, and he had moments of blanking out. That scared me, and I decided I did not want to risk it happening to my child.” (Participant 16).*

*“A friend vaccinated her child about 20 years ago. Her daughter, who had already been walking, suddenly stopped and developed multiple health problems.” (Participant 11).*

*“The anti-vaccine voices are powerful — they sound so real. They cite examples, and some of those side effects cannot be reversed.” (Participant 28).*

#### 3.1.7 Expectation tests and consultations before vaccination

Participants indicated that they would be more willing to vaccinate their children if they could first undergo specific tests or consultations they deemed necessary. In their view, this would rule out contraindications and minimize AEFI risk, thus reducing their fear:

*“Children are usually not tested before vaccination, and that puts me off. Side effects tend to happen when a child is not properly prepared for the vaccine.” (Participant 33).*

*“When doctors do pre-vaccination exams, they should also check for allergies or sensitivities — something could happen because of the vaccine ingredients. I even offered to pay for such tests myself if necessary.” (Participant 24).*

#### 3.1.8 Transfer of vaccine-related beliefs across contexts

The transfer effect is an essential mechanism in forming and reinforcing AEFI fears. When discussing vaccines for a specific disease, parents do not rely on knowledge about that particular vaccine. Instead, they project their beliefs and experiences with other vaccines (e.g., flu or adult vaccines) onto childhood vaccines. For example, one parent discussing routine childhood immunizations referenced adult flu vaccines and general distrust toward vaccines:

*“My friends regret getting vaccinated because they never used to get sick, but after vaccination — one friend who’s a lumberjack and works outdoors in freezing temperatures — suddenly started getting bronchitis and other illnesses he never had before.” (Participant 23).*

### 3.2 Communication with medical staff

#### 3.2.1 Insufficient time and personalization in pre-vaccine screening

An unfavorable evaluation of the organization and quality of vaccination procedures in clinics is a disincentive for positive vaccination attitudes. According to participants, healthcare facilities lack flexible vaccination hours and sufficient time for thorough pre-vaccination medical examinations. Parents described these qualification exams as superficial, assembly-line-like, and inadequately adjusted to individual cases:

*“(…)because it’s just a conveyor belt. vaccinations are only performed in the afternoon on 1 day of the week, and only for 2 h.” (Participant 10).*

*“You have to define what you mean by examining a child because. just glancing at them, a quick look into the throat from above, and listening from a distance — if we call that an examination, then yes, the child was always examined. Because sometimes, that’s exactly what the examination looked like.” (Participant 10).*

*“First and foremost, the child should be thoroughly examined before vaccination, not just a quick interview and assembly-line process... because there’s no time for proper auscultation.” (Participant 26).*

#### 3.2.2 The role of empathy and dialogue in addressing parental vaccine concerns

In addition to identifying contraindications, the qualification exam provides an opportunity to discuss concerns with the doctor. A lack of time or omission of this step contributes to negative parental attitudes toward vaccination. Participants emphasized the need for understanding and empathy from doctors:

*“Doctors themselves make things harder by treating parents dismissively, avoiding conversations, neglecting contact, and adopting a condescending attitude toward parents who have concerns and ask questions.” (Participant 10).*

*“Everyone would feel better if they could just have a conversation — without judgment, without being immediately labeled as an anti-vaxxer. That’s not the case at all.” (Participant 12).*

Parents need access to vaccine information because conversations with clinic staff are often too brief and rushed. Feeling unsupported by the healthcare system, parents begin to search for information on their own — most often online:

*“For heaven’s sake, how are we supposed to know certain things if we are not supposed to read random online forums and unreliable sources? That’s why we ask doctors — but there’s never time for that.” (Participant 10).*

*“Let us not kid ourselves — now Instagram and TikTok are the most popular. I follow accounts like ‘Pan Tabletka’ (Mr. Pill) and a pediatrician who explains things. I feel like they are knowledgeable and trustworthy.” (Participant 18).*

*“The internet, right? Nobody goes to libraries anymore.” (Participant 10).*

Some parents who expressed doubts about vaccines in the doctor’s office reported feeling ridiculed or humiliated:

*“It wasn’t a conversation with arguments; it was a conversation full of accusations and shouting. That discouraged me — I realized I could not even talk to anyone about my concerns. As soon as I said I had doubts or was scared, I was immediately labeled a conspiracy theorist.” (Participant 24).*

Another source of resistance among parents was being presented with consent forms to sign just before vaccination. Parents said they would prefer to calmly review the pros and cons before making a final decision:

*“I have no idea what’s going on because before I finish filling out all the forms, the child has already left the room, and I’m told they are eligible for vaccination.” (Participant 10).*

*“Once, the vaccination nurse attacked me aggressively, forcing me to sign ridiculous forms [stating refusal to vaccinate my child].” (Participant 3).*

#### 3.2.3 Medical dissonance and its role in vaccine hesitancy

Parents noticed that doctors often had differing opinions about vaccinations. These inconsistencies led some to delay or refuse entirely their children’s vaccinations:

*“(...) if our specialists do not agree among themselves, I’d rather wait.” (Participant 20).*

*“We saw several specialists - some said it was okay to vaccinate and gave the green light. Others said the opposite — they said if I want a ‘vegetable,’ I should vaccinate, but they would not sign off on it or give official approval. So now we are stuck, even though I believe vaccines are necessary.” (Participant 26).*

Interestingly, parents who had doubts or refused vaccinations often reported that a doctor, nurse, or midwife had discouraged them from vaccinating or reinforced their negative attitudes toward vaccination:

*“My former pediatrician told me she respected my decision not to vaccinate my children, and that I wasn’t harming them - because if I were, she would intervene.” (Participant 22).*

*“I’ve also met many doctors who were happy about my decision and even patted me on the back, saying they did not vaccinate their kids either. That reinforced my belief.” (Participant 24).*

Some parents feared that vaccination staff lacked up-to-date knowledge. When healthcare professionals were unable to address parents’ concerns due to insufficient information effectively, parents sought answers elsewhere — increasing the risk of exposure to misinformation:

*“The pediatrician, who was quite old, looked at me and asked, ‘Were you sick during pregnancy?’ I said no, and she replied, ‘That’s strange - where did your child’s condition come from then?’ After that, I realized if that’s her level of knowledge, I need to rely on the internet.” (Participant 28).*

*“Even when I go in with my son for vaccination and ask questions, I feel like the doctor does not always fully answer. Sometimes the answers are roundabout — I always leave feeling unsatisfied.” (Participant 16).*

Some parents also criticized the current state of medical education, arguing that it focuses too much on rigid treatment algorithms rather than individualized, holistic approaches:

*“(...) older doctors seem better because nowadays — looking at my eldest daughter, a paramedic — I see how they teach her to follow rigid protocols: symptom A equals action B. They’re not taught to think independently or consider alternative approaches.” (Participant 4).*

Positive interactions with healthcare professionals helped reduce anxiety about vaccination among certain participants, making them more inclined to vaccinate their child:

*“I had a fantastic doctor who did not pressure or try to scare me but simply explained everything. I truly felt that he was there for me. Incidentally, he has a sick child with heart problems, and he vaccinates as much and as often as possible. He even gave me his private phone number and told me to call him anytime or night—and that he would come. That gave me great support, the feeling that if something happens this time, I will not be left alone.” (Participant 25).*

### 3.3 Distrust of the recommended vaccination schedule

The surveyed parents expressed concerns about the number and timing of vaccinations. Some participants questioned the necessity of administering all vaccinations, believing it could lead to excessive strain on children’s immune systems. They also highlighted that the intervals between individual vaccines were too short. They compared this to their vaccination history, noting that they had received fewer vaccines in their lifetime and still enjoyed good health. Their anxiety was further exacerbated by the fact that vaccines were administered to children within the first few months of life:

*“(…) I think that in Poland, the vaccination schedule is, so to speak, overstimulated. I would say that there are just too many vaccines in the first 6 months of a child’s life.” (Participant 12).*

*“I remember my health booklet [record of vaccinations], and there were significantly fewer vaccines back then than there are now. In the first and second year of life, there are just too many.” (Participant 26).*

Creating a rigid vaccination schedule may sometimes be futile, as the parent might not accept it. The lack of flexibility among doctors was a common reason parents decided against vaccinating their children.

*“We have a vaccination appointment tomorrow, and the doctor said we could do pneumococcal and MMR together, but I decided to separate them. First, I’ll vaccinate with one; after some time, I’ll do the pneumococcal vaccine. I will not do both in one visit.” (Participant 23).*

*“Finding a doctor willing to create an individual vaccination schedule for children who missed their infant and early childhood vaccines was impossible. No one was interested in discussing the topic, so I just gave up.” (Participant 24).*

### 3.4 Risk assessment

Some of the respondents did not perceive vaccine-preventable diseases as severe or common. Compared to the potential AEFI, this led them to reject the vaccination altogether. This attitude is reflected in the following statements:

*“I will not vaccinate her against the flu. No, I think there’s too much fear compared to what I could gain.” (Participant 28).*

*“When we saw how these diseases progressed and that they were no longer as dangerous as they used to be, we decided to stop vaccinating our kids—and ourselves.” (Participant 32).*

Parents also questioned whether they should vaccinate their children and expose them to the potential adverse effects of a rare disease. The following statement illustrates how one mother assessed risk before deciding to vaccinate:

*“Meningococcal, maybe yes (…) those are the vaccines after which the side effects are visible and painful, but the disease itself is rare. However, if it does happen, it’s hazardous.” (Participant 3).*

The child’s health status, particularly the presence of chronic illnesses, prompted parents to seek extensive information about vaccines and carefully assess the risks and benefits associated with vaccination:

*“(...) our situation is unique because we have a child with a heart condition who has undergone several cardiac surgeries. So, as parents, we take special responsibility and pay close attention to our child’s well-being.” (Participant 17).*

Parents also evaluated the risks related to how vaccines were administered-whether it was better to give multiple vaccines at once (as with combination vaccines) or to separate them:

*“We do not use all the vaccines, only the essential ones, and we avoid combination vaccines. No, we do not go for those 3-in-1, 4-in-1, or 5-in-1 because if the child reacts, we will not know which vaccine caused it, right? But if we do them separately… I think it’s better. We wait, observe, and if everything is fine, we proceed with the next one.” (Participant 4).*

### 3.5 Conspiratorial thinking/anti-system sentiments

Conspiratorial thinking was frequently a source of doubts regarding vaccinations. Participants were convinced that experts who speak negatively about vaccines are excluded from official discourse and even stigmatized. In the case of doctors, participants believed this could lead to losing their medical licenses. An example of this belief is the following statement:

*“I have many books on this topic [the harmfulness of vaccines], and I also listen to doctors who often lose their licenses afterward.” (Participant 19).*

Participants built their opposing arguments about vaccinations based on the theories promoted by such individuals. These opinions often connected vaccination conspiracies with other unrelated conspiracy theories. One participant expressed suspicion that positive HIV test results might be linked to vaccines:

*“I heard Dr. Luc Montagnier from France — a Nobel laureate — say that some fragment of the HIV was used to create the vaccine, or something like that? And now I’ve heard that many more HIV cases are being recorded in Poland. I do not know if this is the reason, but maybe the tests detecting HIV are somehow linked to how these vaccines were developed. So, are they safe?” (Participant 2).*

Another participant linked some vaccines to planned depopulation efforts:

*“The population is growing too fast. It cannot get too big, so I do not hide the fact that these medications are also, in my opinion, meant to stabilize the population a bit - so there aren’t too many people in the world, and things do not get out of control. So, vaccines are... well, they serve a purpose.” (Participant 14).*

Governments and pharmaceutical companies were also frequent targets of conspiratorial thinking. Participants say these entities are profit-driven, with vaccine production and promotion as part of their business strategy. Some participants also believed that doctors themselves are complicit, more focused on earning money from prescribing certain companies’ vaccines than on patients’ health:

*“The world is corrupt. Everything revolves around money. All kinds of motives guide pharmaceutical companies, so I’ll never truly know the full picture with my level of knowledge.” (Participant 1).*

*“It’s clear that even our so-called experts are on those [payroll] lists - publicly available lists - showing they were paid by pharmaceutical companies, which probably influenced certain decisions made in our country.” (Participant 22).*

Another belief illustrating alleged manipulation was the conviction that vaccine leaflets provided in Poland contain different information than those in other countries. This raised fears that crucial details about potential AEFIs were deliberately hidden in Poland:

*“All the adverse effects listed by the manufacturer — especially in the original leaflets — aren’t translated word-for-word in Polish versions.” (Participant 15).*

Participants also expressed doubts about vaccine ingredients and the alleged lack of quality control. Vaccines were believed to contain toxic substances, which for some parents was reason enough to refuse vaccination altogether:

*“They contain lots of neurotoxins — aluminum, mercury, for example — so in general, I’m against all vaccines.” (Participant 22).*

*“I feel there’s a lack of full control over the composition of vaccines — full control of what’s actually in them. Doctors are saying that even trace amounts of heavy metals are present. Formaldehyde, too, if I remember correctly.” (Participant 4).*

The legally mandated nature of vaccinations in Poland also raised many concerns. Participants noted significant differences in vaccine requirements between Poland and other European and global countries. They pointed out that not all countries enforce compulsory childhood vaccinations. For some, this mandatory nature itself was discouraging:

*“Poland is probably one of just two or three European countries that have a mandatory vaccination system, right? Most Western countries — the more rational ones — allow more freedom of choice.” (Participant 17).*

*“There’s no requirement to vaccinate in England, right? And they manage somehow… In Poland, they force you, and everyone thinks that means it’s good. But if it’s not required there, what does that mean? That it’s bad? Does not make sense.” (Participant 19).*

Another argument raised by participants fueling their hesitation toward vaccines was the lack of comparative studies on the health of vaccinated versus unvaccinated children. Some even referred to alleged studies claiming better health outcomes for unvaccinated children:

*“They vaccinate newborns right away, but they never compare the health of vaccinated children with unvaccinated ones - even though other countries do this.” (Participant 19).*

*“I’m talking mainly about Japan. They have very advanced research and confirmed data. Similar studies were also done in Australia, comparing vaccinated children up to age two with unvaccinated ones - and the unvaccinated ones were healthier.” (Participant 15).*

Parents were also concerned that some vaccines, especially COVID-19 vaccines, were rushed to market without proper testing and long-term observation:

*“COVID vaccination — especially for children — based on everything I’ve learned, nobody has convinced me otherwise. These vaccinations for children were completely unnecessary, pointless, and carried high risks because those vaccines were never fully tested.” (Participant 6).*

*“I did not vaccinate my children against COVID. It was too new. Unproven. I vaccinated myself, but not my kids.” (Participant 11).*

Several participants recruited through Facebook groups reported engaging with vaccine-related content primarily as passive observers. Rather than actively contributing to discussions, they often monitored the dialogue and drew their conclusions based on the tone, arguments, and shared experiences of others. This form of silent participation allowed them to process information at their own pace, without exposing themselves to potential judgment or confrontation, especially in groups where polarized views and emotional rhetoric were common:

*“I’m glad I had the chance to share this story with someone, and I know that it might carry more weight than if I were to open up in an online group. Like I said, those spaces are usually very polarized - either strongly pro-vaccine or anti-vaccine-and there’s no real dialogue. There’s no room for discussion, and that’s also a problem. If it’s a pro-vaccine group encouraging immunization, they should not attack people who have doubts or who have had negative experiences. That’s why I stopped participating in those groups.” (Participant 25).*

### 3.6 Organizational and financial issues

Financial barriers related to vaccination emerged in several interviews with parents, particularly those closer to the accepting end of the vaccine hesitancy continuum. The study participants pointed out that the list of vaccines covered by public funds should be expanded and updated. This particularly applies to highly combined and meningococcal vaccines, which parents must pay for out of pocket. If parents lack financial resources, they are unable to access these vaccines. In Poland, vaccine reimbursement primarily covers vaccines included in the National Immunization Program as mandatory vaccinations. This program is based on data regarding the current epidemiological situation of infectious diseases and is adapted to the state’s financial capabilities. Parents can choose to fulfill the vaccination requirements using publicly funded vaccines or bear the cost of recommended vaccines, including highly combined vaccines and those with a broader spectrum of protection. For example, the 13-valent pneumococcal vaccine is covered by public funds, while the 20-valent version requires an out-of-pocket payment. The inability to use highly combined vaccines exposes children to multiple injections. This presents a challenge not only for clinic staff but, most importantly, for the child. In many cases, it is not possible to administer all required vaccines in a single visit, leading to additional appointments to complete the vaccination schedule. As a result, children experience delays in their vaccinations. Parents emphasize that this situation puts them in a difficult position-if they want to ensure the best protection for their child (such as opting for highly combined vaccines, meningococcal vaccines, or those offering broader coverage, like the 20-valent pneumococcal vaccine), they must pay for combination vaccines themselves.

*“If these additional vaccines were reimbursed, parents could decide. I’ve come across cases where parents could not afford to vaccinate. They wanted to, but they just could not afford everything.” (Participant 26).*

*“I did not vaccinate against meningococcal disease because, financially, it just did not work out at the time.” (Participant 21).*

*“But honestly, if I had the financial means, I would have gone for the combination vaccines. From what I know, they are recommended for children in high-risk groups, so I assume they must be safe, right?” (Participant 21).*

Another reason some parents did not bring their children for vaccinations was the lack of recommendations from medical staff regarding specific vaccines.

*“Just like you said, we were not offered certain vaccines. There were not any concerns, but honestly, my child has never been vaccinated against the flu. Not even once.” (Participant 31).*

Additionally, parents mentioned that they did not receive reminders from healthcare facilities about upcoming vaccination appointments.

*“(…) I do not always keep track of the vaccination calendar. When kids are younger, you pay more attention but do not monitor it as closely as they grow older. There are so many other things to deal with besides vaccinations. If someone from the clinic does not call me to remind me that my child has turned 10 and needs a specific vaccine, I will not really… I will not pay much attention to it. If I remember, I’ll check it myself and maybe contact the clinic.” (Participant 10).*

## 4 Discussion

Our study provides insights into the attitudes of Polish parents toward childhood vaccinations. The collected material allowed for identifying six main issues (Table 4) that determined parents’ decisions regarding vaccinating their children. The most frequently reported concern was AEFI, followed by problematic communication with medical staff, distrust toward the recommended vaccination schedule, risk assessment, conspiracy theories, and organizational and financial issues.

The COVID-19-related vaccinations were deliberately omitted in the prepared topic guide. The primary focus was on mandatory vaccinations within the childhood immunization schedule. However, the in-depth interview format allowed for the topic to be included and developed if the interviewee introduced it. It was exciting to observe whether and to what extent this topic would emerge, as it could indicate how strongly the pandemic and COVID-19 vaccines influenced the current discourse on vaccinations. Previous studies suggest that such an impact exists (26, 27).

During the interviews, all parents — without exception — referenced their experiences from the pandemic, particularly regarding their knowledge about the safety and side effects of COVID-19 vaccines. They used this as a justification for their current views on vaccines and their hesitancy about vaccinating their children.

The reasons for vaccine hesitancy differ between countries, proving that vaccine hesitancy is complex and context-dependent. This also highlights the importance of locally identifying such factors and designing tailored interventions to address them (28).

Our study confirms, consistent with existing quantitative research, that fear of side effects is the most common reason for parental doubts about childhood vaccinations (19). There is also a distinctive understanding of the term AEFI among parents. They typically do not associate AEFI with mild reactions like soreness at the injection site or a temporary fever. Instead, AEFI is understood as a severe post-vaccine reaction, usually neurological (with effects potentially emerging after a more extended period) or a severe allergic reaction (occurring immediately or shortly after vaccination). Similar findings — showing fear of vaccine side effects as a demotivator — have been reported in studies from Italy (29), France (30), the USA (31), and China (32).

As in our study, the fear of neurological complications is one of the most common specific concerns about severe side effects. A review of the literature indicates that the fear of autism is particularly significant (33). This stems from the widely discredited publication that falsely linked the MMR vaccine to autism (34). Despite conclusive scientific evidence debunking this connection, the false belief continues circulating among vaccine-hesitant parents (35).

Parents of children with chronic illnesses are susceptible to the possibility of AEFI. In their desire to protect their children, they emphasize the need to thoroughly research vaccines before proceeding. They believe the risks and complications associated with diseases are not evenly distributed across society. They understand that their children are more vulnerable to complications from vaccine-preventable diseases (VPD), but due to fear of side effects, they often choose to refuse or delay vaccinations. Similar findings were observed in a post-pandemic study in Italy, where nearly one-third of parents of children with chronic illnesses expressed significant concerns about vaccine side effects. The PACV (Parent Attitudes about Childhood Vaccines) indicator showed that 23.2% of these parents exhibited vaccine hesitancy (36).

Another issue raised by participants was the perceived lack of an efficient compensation system for vaccine injuries or health damage following vaccination. They referenced systems in countries like the United States, where evident, uncontested compensation programs exist for vaccine-related harm (37). Poland’s Protective Vaccination Compensation Fund Act was enacted shortly before our study — on January 27, 2022. Most respondents were unaware of its existence. According to the law, until the end of 2022, compensation was only available for adverse effects following COVID-19 vaccinations. From 2023 onwards, compensation covers all mandatory vaccinations (38).

We also explored sources of information about vaccines and AEFI. Both our interviews and other studies show that negative opinions about vaccines were often linked to direct or indirect contact with people who claimed to have been harmed by vaccines (29, 39). Media stories influenced some parents, while others referred to accounts from their networks or experiences. Interestingly, although quantitative Polish studies (40) — and studies in other countries (36, 41) — indicated that HCPs (healthcare professionals) are the primary source of information, parents ultimately make decisions based on conversations with other parents, friends, or online sources. Literature and our research confirm that mothers’ beliefs are particularly influential, often shaped by personal experiences and views shared within their social circles (30, 42). Mothers are more than twice as likely as fathers to take responsibility for their children’s healthcare (43).

A scoping review of parental attitudes, motivations, and barriers to childhood vaccinations in Poland between 2014 and 2024 showed that women comprised the majority of participants in this type of research, with participation rates ranging from 56 to 100% (19). Studies also show that mothers develop trust in doctors when they take the time to thoroughly discuss vaccination-related issues, take parental concerns seriously, demonstrate expertise, and provide clear, satisfactory answers (44). Our participants also emphasized that the voices of other mothers sometimes outweigh professional medical advice.

This finding ties into another critical issue revealed by our research — the quality of communication and cooperation with healthcare providers responsible for qualifying and administering vaccines. Parents in our study expressed that HCPs should devote more time to informing them about the benefits of vaccination and addressing their concerns. Approaching hesitant parents with empathy is particularly important. Other qualitative studies also show that negative parental experiences — such as poor relationships or unsatisfactory communication with healthcare providers — can discourage vaccination (45).

Two main factors influence communication problems: the doctor’s personality and communication skills and the vaccination system’s organization. Parents observed that some doctors — when asked about side effects — could be dismissive, rude, or condescending, immediately labeling concerned parents as anti-vaxxers. On the other hand, parents noted that short appointment times, routine procedures, and the need to complete paperwork during the visit effectively prevented meaningful conversations.

This is particularly important because many studies show that improving communication with HCPs could be one of the simplest and most cost-effective ways to change parental attitudes and increase childhood vaccination rates. An example is a study among primary care doctors in the United States, where the most effective communication strategies involved personal statements from doctors about what they would do for their children and their personal experiences with vaccine safety among their patients (46).

Another critical factor influencing vaccine hesitancy is the inconsistent information from healthcare providers. All HCPs must adhere to current scientific knowledge and recommendations. Our study revealed that parents sometimes receive conflicting advice — for example, from the vaccinating doctor and a specialist in another field (particularly pediatric neurologists). In such cases, the vaccinating doctor finds persuading parents to proceed with immunization challenging when another specialist provides contradictory advice.

This situation is well-documented in research conducted in Austria and Germany, where vaccine hesitancy among general practitioners and pediatricians was linked to practitioners being involved in homeopathy and non-evidence-based medicine (47). The literature also shows that vaccine hesitancy is more common among nurses than doctors. Doctors tend to worry more about vaccine efficacy against evolving pathogens, while nurses focus more on potential side effects in children (48).

Some parents in our study also questioned the competence of vaccination staff, believing they lacked sufficient knowledge. Other research confirms this concern. A study to identify factors to strengthen vaccination systems in five European countries (Lithuania, Romania, Slovakia, Slovenia, and Spain) highlighted insufficient HCP training as a barrier to effective measles vaccination (49). Although Poland was not mentioned in this report, our participants repeatedly cited the incompetence of some doctors performing pre-vaccination qualifications.

The doctor-parent relationship is crucial, which is why parents in our study adopted the tactic of seeking out a friendly doctor — someone who had the right personality and confirmed their doubts or even supported their anti-vaccine stance. Some parents reported switching to private healthcare facilities to find more flexible doctors who respected their opinions. Similar findings were reported in a French study where one mother described her ideal doctor as “I found a shoe that fits” (30).

The significant importance of the doctor-parent relationship is also confirmed by positive opinions, where openness, the time dedicated to the patient, and the ability to reassure can convince even skeptical parents to vaccinate their child. It is also worth noting that doctors should pay special attention to parents vaccinating their child for the first time. This is due to so-called inertia, which we also observed in our research. This means parents with more than one child usually ask many questions about vaccinations when immunizing their first child. Still, subsequent children follow their previous decisions routinely to save time and worry (30).

The need to improve communication is signaled not only by parents but also by doctors themselves. A study conducted among HCPs in England and France highlights the need for further training in communication to address patients’ doubts effectively (50). Unfortunately, many HCPs feel unprepared to answer parents’ questions regarding vaccinations for various reasons, including a lack of training in evidence-based communication strategies (51). When analyzing the statements of our respondents, we also noted that ineffective communication can be a barrier to parents giving consent to vaccinating their children. Parents expect clear messaging, active listening from HCPs, and an empathetic attitude. Therefore, training in vaccination-related communication is necessary to meet the needs of a changing society and the environment in which the immunization program is implemented.

A crucial issue for the respondents was the vaccination schedule. They expressed concerns about whether vaccinations start too early, whether there are too many in a short period, or whether multiple vaccines should be administered in one visit. These concerns are not unique to Polish parents and affect those who refuse vaccinations and comply with immunization recommendations (52, 53). Such problems are exacerbated by widely available information on vaccination schedules in other countries. Parents compare these schedules and question the necessity of strictly following their own country’s guidelines, leading to requests for modifications in the vaccination schedule. This is confirmed by other studies among pediatricians in Washington State, where 77% reported that their patients sometimes or often requested an alternative vaccination schedule (54).

Supporters of alternative schedules aim to reduce the risk of adverse effects, which they believe may be linked to “immune system overload” caused by exposure to too many antigens (41, 55) and the fact that vaccinations are administered to very young children (33, 39, 56, 57). Similarly to our study, Lyndal Bond and Terry Nolan (56) confirmed that non-vaccinating individuals believe their children are unlikely to experience severe complications from diseases because they have a healthy immune system.

When making vaccination decisions, parents assess risks, calculating whether the side effects of vaccines could be severe, whether vaccine-preventable diseases are hazardous (30, 39), and whether these diseases are genuinely prevalent (30). For example, illnesses such as measles, mumps, and rubella were not considered severe or life-threatening in one of the analyzed studies. Most mothers were familiar with these diseases, had personal experiences, and remembered them as mild (56). Some parents even believe contracting certain diseases is unlikely or impossible since they are virtually non-existent (30).

Other studies conducted in Poland have observed that real or perceived ties between policymakers, doctors, and the pharmaceutical industry are an essential concern. Media reports portraying clinical research, drug availability, and pricing in a negative light—regardless of their objectivity—raise parental fears (26). Research by Klimiuk et al. on comments about vaccinations in Polish social media has shown that conspiracy theories and misinformation are the most common forms of communication (58).

Another source of information for our study participants was vaccine manufacturer leaflets. Many respondents noted differences in the content of vaccine leaflets distributed in Poland compared to other countries. This may lead to the formation of conspiracy theories. A study examining opinions on transitioning to electronic vaccine leaflets highlighted differences in leaflet content across countries. While the study did not focus specifically on conspiracy theories, such discrepancies could be exploited by anti-vaccination movements to spread misinformation. It is important to note that differences in vaccine leaflets between countries result from varying regulatory, linguistic, and cultural requirements rather than hidden agendas by manufacturers or governments (59).

As stated by SAGE, fear of injection pain is also a determinant of VH in the pediatric population (5). This is particularly evident in studies on attitudes toward influenza vaccination. A study conducted in Bologna found that nearly 72.2% of parents stated they would not vaccinate their children against influenza. However, 40.2% of them changed their minds after learning about the availability of a needle-free vaccine. The primary reason for this shift was children’s fear of needles (60). A 2018 systematic review assessing the prevalence of needle fear found that most children exhibited this fear, with an estimated prevalence ranging from 20 to 50% in adolescents, decreasing with age (61). Interestingly, one study found that parents of children aged 12–17 wanted to involve their children in healthcare decision-making and one of the reasons children refused vaccinations was fear of needles (62).

This contrasts with our observations, where respondents rarely mentioned fear of injections as a significant deterrent to vaccination. Nevertheless, some respondents opted for combination vaccines to minimize the number of injections. Parents emphasized, however, that their primary concern was safety, as high-combination vaccines contain fewer additional substances, such as preservatives or residual compounds from the production process (e.g., formaldehyde). The reduced number of injections was considered a secondary issue.

Unfortunately, parents who choose highly combined vaccines for children up to two must cover the full cost in Poland. Our study showed that financial barriers could hinder access to recommended vaccines, potentially leading to lower overall vaccination rates in Poland. This issue also applies to meningococcal vaccinations. Similar challenges exist in many European countries, where out-of-pocket payment for influenza vaccines is a barrier to achieving higher vaccination rates (49). For example, in China, respondents were more likely to get vaccinated when vaccines were free or partially covered by public funds (32).

## 5 Limitations

This study provides new insights into parental concerns regarding vaccinations but has certain limitations. First, based on Facebook groups and snowball sampling, the recruitment strategy may have introduced selection bias (the sample was not representative), potentially resulting in an overrepresentation of individuals with strong views and a greater willingness to express them. Consequently, the sample may reflect more critical attitudes toward vaccination than the general population. Furthermore, there is a risk of recall bias resulting from inaccuracies in remembering past experiences or events. Additionally, self-report bias is possible, as the analysis was based on data provided by the study participants without the ability to verify it against vaccination records. Since most parents did not have their children’s vaccination records with them, some could not answer specific, detailed questions with complete certainty. This issue was particularly relevant for parents of older children who had not dealt with vaccination-related matters for a long time. Although we achieved data saturation in this study, it is possible that we did not reach participants who could have provided new, significant information.

## 6 Implications for practice and research

Future research should consider employing more diversified and systematic recruitment strategies to address the limitations identified in this study and minimize selection bias. To reduce the risk of recall bias, researchers may prioritize including parents of younger children navigating vaccination schedules or incorporating verified vaccination records when possible. Accessing official health documentation or encouraging participants to consult vaccination booklets during data collection could improve accuracy. Additionally, to mitigate self-report bias, future studies might triangulate self-reported data with other sources, such as health records or survey data, while ensuring participants’ anonymity and creating a neutral interview environment. Finally, expanding recruitment efforts-through more extended data collection periods, outreach in community settings, or participatory approaches-may help capture the views of harder-to-reach populations and uncover perspectives not represented in this study.

Our findings underscore the practical need for targeted training of HCPs in the content and delivery of vaccine-related communication. Training programs should incorporate specific evidence-based strategies to effectively address parental VH, such as motivational interviewing, active listening, and empathy-based dialogue. Furthermore, these efforts should not be limited to professionals directly administering vaccines but extended to all HCPs who engage with parents and caregivers, ensuring consistent, evidence-informed messaging across the healthcare system.

Structural changes within healthcare institutions are also necessary to support these improvements, including allocating dedicated time and resources that enable HCPs to engage in continuous education and interprofessional training.

Policy interventions should be considered to address the financial barriers identified by participants-particularly those related to broader-spectrum, highly combined, or recommended vaccines such as those against meningococcal disease. Expanding the national immunization program to include these vaccines or offering them at reduced cost through partial reimbursement schemes could significantly improve access. Additionally, implementing targeted subsidies for families with lower income or providing such vaccines free of charge during specific time-limited public health campaigns may help reduce inequalities in access to what parents perceive as the “best protection” for their children. These measures could enhance vaccine uptake among hesitant but generally pro-vaccination parents motivated but constrained by cost.

## 7 Conclusion

Our study offers a novel contribution by employing a qualitative approach to explore parental attitudes toward childhood vaccination in the post-pandemic context in Poland. Unlike most existing research in this area, which has predominantly relied on standardized tools such as surveys and quantitative statistical analyses, our study seeks to provide a deeper, more nuanced understanding of vaccine hesitancy through in-depth interviews. We focus on parents’ lived experiences and individual reasoning to identify the underlying causes of vaccine hesitancy in a country where skepticism and distrust toward vaccination have gradually increased since the political transition in 1989, and where many perceive the COVID-19 pandemic as a significant catalyst that has further deepened these concerns. This qualitative perspective allows us to capture the complexity of vaccine decision-making processes often obscured in large-scale quantitative studies. We found that concern about AEFI was the most significant reason for parental hesitancy regarding childhood vaccinations. The second key factor was the attitude of HCPs when interacting with these parents. In many cases, the response of Primary Healthcare Clinicians (PHCs) played a crucial role in determining whether parental concerns about AEFI were alleviated or reinforced, leading to a decision against vaccination. Additionally, organizational and financial barriers discouraged parents from vaccinating their children. Participants expressed a desire for a more individualized approach to the vaccination schedule and a reduction in financial barriers.

## Data Availability

The raw data supporting the conclusions of this article will be made available by the authors, without undue reservation.
